# The Combinatorial Fusion Cascade to Generate the Standard Genetic Code

**DOI:** 10.3390/life11090975

**Published:** 2021-09-16

**Authors:** Alexander Nesterov-Mueller, Roman Popov

**Affiliations:** Institute of Microstructure Technology, Karlsruhe Institute of Technology (KIT), 76344 Eggenstein-Leopoldshafen, Germany; roman.popov@axxelera.com

**Keywords:** origin of genetic code, prebiotic chemistry, time order of canonical amino acids

## Abstract

Combinatorial fusion cascade was proposed as a transition stage between prebiotic chemistry and early forms of life. The combinatorial fusion cascade consists of three stages: eight initial complimentary pairs of amino acids, four protocodes, and the standard genetic code. The initial complimentary pairs and the protocodes are divided into dominant and recessive entities. The transitions between these stages obey the same combinatorial fusion rules for all amino acids. The combinatorial fusion cascade mathematically describes the codon assignments in the standard genetic code. It explains the availability of amino acids with the even and odd numbers of codons, the appearance of stop codons, inclusion of novel canonical amino acids, exceptional high numbers of codons for amino acids arginine, leucine, and serine, and the temporal order of amino acid inclusion into the genetic code. The temporal order of amino acids within the cascade is congruent with the consensus temporal order previously derived from the similarities between the available hypotheses. The control over the combinatorial fusion cascades would open the road for a novel technology to develop artificial microorganisms.

## 1. Introduction

The origin of the standard genetic code (SGC), more specifically, the codon distribution over canonical amino acids is one of the fundamental scientific problems. Mastering the molecular apparatus for generating artificial code would enable novel efficient microorganisms for scientific, medical, and industrial applications.

Koonin distinguished three major theories—error minimization, coevolution, and stereochemical—that strive to explain the regularities in the standard genetic code [1]. If the origin of the genetic code is considered in the scope of protein evolution, then this “top-down” approach leads to the consensus about the gradually evolved SGC from some initial primordial code. For example, P. Higgs, using the advanced error minimization model, showed that his four-column theory with a primordial code consisting of non-biologically synthesized amino acids Gly, Ala, Asp, Glu, and Val fit perfectly with a row of predictions from the coevolution theory [2]. S.E. Massey proposed that error minimization of the SGC could arise via the genetic code expansion, facilitated by the duplication of genes encoding charging enzymes and adaptor molecules [3,4].

The theory of coevolution was proposed by Wong in 1975 [5]. It is based on the idea that the genetic code originally consisted of a few amino acids—precursors, which occupied all available coding triplets and were subsequently replaced by their products—late amino acids. Wong defined one major center of amino acids consisting of Glu, Asp, Ala, Ser, and Gly, from which 11 other amino acids evolved, and the two minor centers of Phe-Tyr and Val-Leu. This idea turned out to be very productive as it stimulated research on synthetic pathways from prebiotic chemistry to microbial metabolism, which have made significant progress to date [6,7]. A detailed description of the coevolution theory and its variations can be found, for example, in the works of Di Gulio [8] or Wong et al. [9]. A controversial issue in coevolution theory is the principle of codon transfer from precursors to products (see discussions between author Di Giulio and reviewers Koonin, Knight and Higgs [10]). Wong himself, faced with the fact that Leu and Arg each occupy as many as six coding triplets, but do not belong to the major center, assumed: “To acquire and retain a high plurality, like Leu and Arg, the amino acid had to be both early in arrival and inert in reactivity” [5].

Remaining within the framework of this “top-down” approach, other arguments can be made in favor of the gradually evolving genetic code. Bases guanine G and cytosine C prevail in the acceptor stem of modern tRNA, and therefore the amino acids associated with the codons from these bases appeared earlier in the code [11,12]. However, the very unstable canonical amino acid arginine does not fit into this concept. The late branching of Class I aaRS (aminoacyl-tRNA synthetases) implies an early origin of the amino acids coded by the Class II aaRS [13,14]. The gradual expansion of the coding space as GC–GCA–GCAU genetic code was proposed by Hartman and Smith [15]. Kubyshkin and Budisa supported this hypothesis, demonstrating the correlation of this scheme with the hierarchy of the protein folding [16].

The anticodon binding domain typically provides added specificity for tRNA substrate interactions with aaRS [17,18]. This feature is missing by LeuRS, SerRS, and AlaRS, which may point out an early origin of the respective amino acids in the genetic code.

The modern stereochemical theory profits from the progress in aptamer technology and does not need the assumption about the gradual entry of amino acids into the code. It focuses on identifying the mechanisms of selective interactions between amino acids and their codons, or anticodons [19,20,21]. Yarus et al. reported statistically significant affinity for the interactions between Arg, His, Ile, Phe, Trp, and their cognate triplets [22]. These results were interpreted as evidence of the existence of an early stereochemical era during the code evolution [23].

The presence of only a portion of the 20 canonical amino acids in abiotic synthesis experiments, for example, in Miller’s experiment [24] implies that amino acids were added to the SGC during its evolution. Although experimental conditions of such experiments such as the composition of gases have been criticized [25] and there are no proven connections with the time when amino acids entered the code and their appearance on Earth, Miller’s results are widely used as key evidence for the gradually evolving genetic code [12,26].

Today, the assumption about the gradually evolving SGC has taken on the status of a postulate. However, this postulate fulfills a constructive role in reconciling modern trends in the science of the code origin: while the stereochemical theory develops a molecular base for the primordial code, the error minimization, and coevolution theories should complementarily describe its evolution to the SGC.

Meanwhile, there are reasons to believe that the origin of the SGC may not relate to the evolution of proteins. Koonin and Novozhilov argued that “attempts to decipher the primordial stereochemical code by comparative analysis of modern translation system components are likely to be futile” [23]. Since the boundary between the primordial code and the code that should have developed adaptively could not be drawn, it is possible to extend this statement to most of the genetic code.

This assumption is not new. According to Woese et al., the evolution of aminoacyl-tRNA synthetases certainly influenced the formation of the modern translation mechanisms, but did not shape the codon assignments [27]. The pioneers of the quantitative metric to measure the robustness of codes to error, Haig and Hurst, believed that “the code could acquire its major features before the evolution of proteins [28].” These ideas were put forward over 20 years ago, but have not received much more attention. Against the background of the rapid development of DNA sequencers since 2000, it was first more important to understand how the genomes and proteomes of the most archaic microorganisms relate to the genetic code [29]. This trend has contributed to the intensive development of the theory of coevolution and error minimization in the scope of protein evolution.

The outstanding work of Vetsigian et al. has given a new perspective on the origin of the code, focusing on the issue of its universality [30]. The authors concluded that “horizontal transfer of genes and perhaps other complex elements among the evolving entities [a dynamic far more rampant and pervasive than our current perception of horizontal gene transfer], is required to bring the evolving translation apparatus, its code, and by implication the cell itself to their current condition.” In other words, the emergence of a universal code would be possible as a result of the coexisting competing entities with a horizontal fusion of their molecular apparatus.

The recent discovery of the fusion rules integrated into the SGC led to a simple and mathematically exact description of the codon distribution over canonical amino acids [31,32]. According to these rules, the modern genetic code arose from the fusion of dominant and recessive protocodes, which initially competed for the same triplets. It was concluded that almost all canonical amino acids were already involved in the reproductive apparatus of coexisting protocodes. In this paper, we consider the combinatorial fusion of competing entities in the scope of the combinatorial fusion cascade. We write out combinatorial fusion rules that are objective properties of the genetic code, discuss their rationale, and focus on how combinatorial fusional cascade could exist.

## 2. Combinatorial Fusion Cascade and Its Formalism

**Figure 1:**
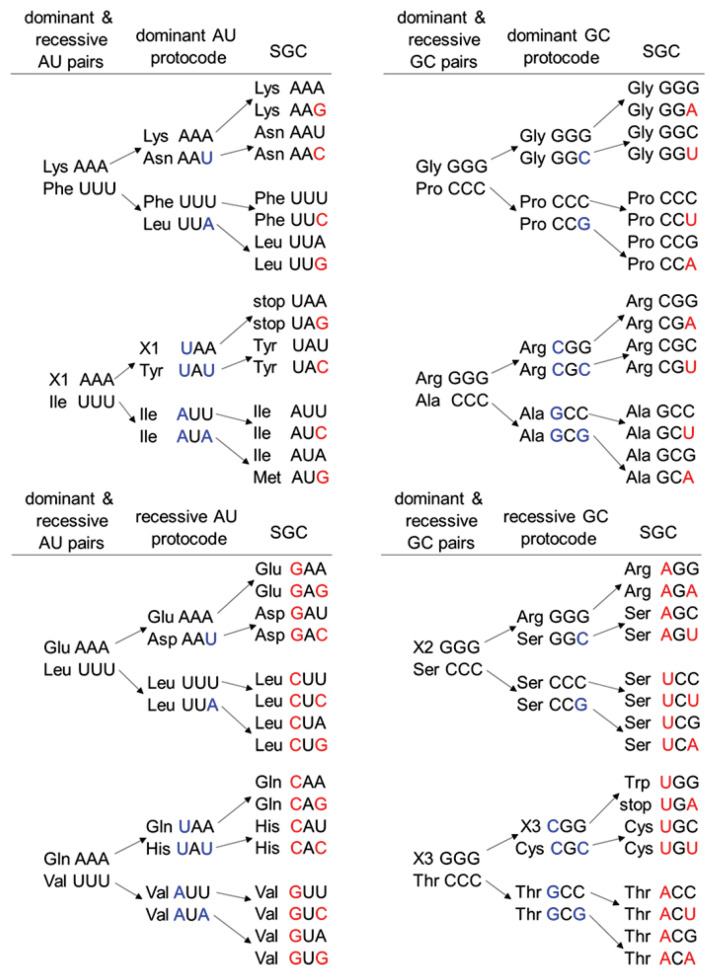
Combinatorial fusion cascade of the canonical amino acids leading to the codon assignments in the SGC. The blue letters indicate the fusion rules for the dominant and recessive AAA/UUU- and GGG/CCC-pairs to the protocodes. The red letters indicate the fusion rules for dominant and recessive AU- and GC-protocodes to the SGC. The fusion pattern is identical for all amino acids: The third position changes in the codons of the dominant entities. The first or the first and the third positions change in the codons of the recessive entities.

Figure 1 suggests that the fusion of protocodes into standard genetic code was part of a larger event that we call the combinatorial fusion cascade. The combinatorial fusion cascade started with coexisting pairs of amino acids. Each of these pairs had complementary monobasic codons AAA/UUU or GGG/CCC. By analogy with protocodes, these pairs were also divided into dominant and recessive ones, depending on the efficiency of their ancient reproduction mechanism. The terms “dominant” and “recessive” are borrowed from classical genetics and refer to the fact that the dominant entities do not change their initial codon-amino acid assignments after the fusion. In contrast, the recessive entities acquire new triplets.

The transition from the initial pairs to the protocodes consisted of the expansion of the initial monobasic triplets with complementary bases (indicated with blue letters in Figure 1). Therefore, the dominant pair Gly/Pro received additional codons GGC and CCG, in which there were substitutions by C or G in the third position, respectively. In the case of the recessive pair Arg/Ala, the same substitutions occurred in the first or the first and the third positions. As a result, Arg received two codons CGG and CGC, and Ala two codons GCC and GCG. The recessive Arg/Ala pair gave up their original codons in favor of the dominant Gly/Pro pair. This example illustrates the formation of the dominant GC-protocode as an intermediate stage to the SGC.

This fusion of the initial pairs to the dominant and recessive protocodes is summarized by the following rules:

Rule 1: The second-position bases do not change in any code.
Rule 2: C and G as well as U and A are exchangeable only in the third position in the dominant initial pairs.
Rule 3: C and G as well as U and A are exchangeable either in the first position or simultaneously in the first and third positions in the recessive initial pairs.

Combinatorial fusion rules of the protocodes to the SGC have the same pattern: dominant codes retain their original codons and have a single mutation at the third position. Combinatorial fusion rules for codon triplets in the protocodes state (Figure 1, red letters) have the form:

Rule 1: The second-position bases do not change in any code.
Rule 2: A and G as well as U and C are exchangeable only in the third position in the dominant protocodes.
Rule 3: A and G as well as U and C are exchangeable either in the first position or simultaneously in the first and third positions in the recessive protocodes.

The fusion rules for the dominant protocodes correspond to the most spontaneously occurring mutation types (A -> G and G -> A as well as C -> U and U -> C) in the third codon (1st anticodon) position noted by Crick shortly after the publication of the code table [33]. This wobble position is occupied by a modified base that is part of the universal genetic code and was probably present in the last universal common ancestor [23]. In contrast, positions 1 and 2 are devoid of mutations in the case of the dominant protocodes. According to Copley et al., these regularities include strong correlations between the first base of codons and the precursor from which the encoded amino acid is synthesized and between the second base of codons and the hydrophobicity of the encoded amino acid [34].

The fusion rules for the recessive protocodes are based on the same mutations, but in the 1st position or in positions 1 and 3. Having identified this fact from the fusion cascade, Nesterov-Mueller et al. suggested that such combinations could be induced within the framework of the kissing hairpin geometry by means of complementary codons [32]. Attempts to explain the emergence of the genetic code from complementary tRNA hairpins were also undertaken by Rodin and Ohno [35]. These kissing hairpins served as proto-tRNAs carrying two amino acids and were a structural element of the protocodes. Hairpins in recessive protocodes had a significantly lower concentration than hairpins in dominant ones. Therefore, they were inhibited by the dominant hairpins and occupied the remaining free combinatorial combinations after the fusion event, which correspond to the fusion rules for the recessive protocodes.

By analogy, we assume that the concentration of the dominant initial pairs in the cascade was higher than in recessive ones, which could be determined by different lengths of linear fragments of complementary RNAs. The initial pairs of triplets had a relatively simple organization including only one hydrophobic and one polar amino acid. At the same time, they could reproduce themselves and participate in the competition with other initial pairs. Most likely, the combinatorial fusion cascade was more extensive and contained other pairs with non-canonical hydrophobic and polar amino acids. However, the reconstruction of the combinatorial cascade from the standard genetic code, according to the fusion rules, led to exactly eight initial pairs of amino acids (total of 16 amino acids). This is equal to the number of amino acids in the doublet codon code proposed by Copley et al. based on the possible synthesis pathways of amino acids from α-keto acid precursors covalently attached to dinucleotides [34]. At the same time, the bases and phosphates of the dinucleotide are proposed to have enhanced the rates of synthetic reactions, leading to amino acids.

Individual parts of combinatorial fusion rules can be reformulated within the framework of group theory. However, we do not believe that group theory is a suitable formalism for describing the combinatorial fusion cascade. The idea of using group theory to explain the origin of the genetic code appeared in the 60s, immediately after the publication of the code table [36]. Afterward, several mathematical approaches to the genetic code in terms of symmetry properties have been developed [37,38,39]. According to D.L. Gonzalez et al. [40], the problem with such descriptions was the difficulty in providing a biological interpretation. A deeper reason for this is that symmetries derived from group theory describe conservation laws in conservative systems. However, the genetic code is not a conservative system, neither in terms of volume nor in terms of energy or mass transport. Consequently, the group theory approach, although it can exactly describe certainly not random symmetries of the SGC and their breaking, could not lead to its ultimate goal—conservation laws and the conclusion about the coexisting protocodes with identical triplets. In contrast, combinatorial fusion rules express this intrinsic fact of the SGC in an explicit and probably the simplest form.

## 3. Results and Discussion

### 3.1. Entering Amino Acids into the Combinatorial Fusion Cascade and Codon Assignment in the SGC

Using Figure 1, the development of the genetic code can be represented in two stages: The entrance of the bases U and A or G and C into the monobasic codons and then the fusion of the protocodes to the SGC. These processes obey a simple mathematical description that is uniform for the entire cascade.

This separation of SGC visually highlights the already known properties of the code and reveals features that were not visible in other representations of the code, for example, in the original tabular form. The formation of an even number of codons for canonical amino acids is a direct consequence of the combinatorial fusion cascade. Ideally, each amino acid from the initial pairs should have four codons. Deviations from four codons (two or six codons per amino acid as well as an odd number of codons) are associated with the entry of new amino acids or the disappearance of old ones.

The capture of new amino acids into the cascade led to a reduction in the number of codons to two per amino acids. At the same time, the new incoming amino acids had similar properties to those that they replaced. For example, aspartic acid (Asp) acquired the codon AAU derived from the AAA of glutamic acid (Glu). In the dominant AU-protocode, the hydrophobic amino acid phenylalanine (Phe) was substituted by the hydrophobic amino acid leucine (Leu). Thus, this transition of Leu from the recessive AU-protocode resulted in the six codons.

The amino acids arginine and serine (Ser) also have a maximum number of six codons. According to the combinatorial fusion cascade, these amino acids acquired an additional two codons after the exclusion of the amino acid X2 from the recessive protocode. It is noteworthy that the division into protocodes showed that each protocode used one positively charged amino acid: dominant AU—Lys, recessive AU—His, dominant GC—Arg, and recessive GC—Arg (X2). These positively charged amino acids may significantly contribute to the specific interactions between negatively charged RNAs and antient peptides. Most likely, the lost X2 was a positively charged amino acid and its substitution occurred with arginine, which carried a positive charge and had the same cognate codon GGG in the dominant protocode. A candidate for X2 could be the guanidinooxy analogue of L-arginine—the weaker base canavanine (Cav). Cav is found in some legumes. It easily integrates into proteins instead of arginine and is toxic to many organisms [41]. The role of Cav in microorganisms, especially in nitrogen fixation, is still far from being understood [42].

The appearance of an odd number of codons (three stop codons, one for methionine (Met), and one for tryptophan (Trp)) refers to the later stabilization of the SGC after the combinatorial fusion. Met and Trp are the most poorly represented amino acids in the genomes. Trp entered the code after the fusion of the protocodes occupying one of the initial stop codon UGG. Similar substitutions were also observed for the stop codons in the SGC. Non-canonical amino acid pyrrolysine (Pyl) occupied stop codon UAG in some prokaryotes, which was necessary to develop the methane metabolism [43,44]. The free stop codon UGA was adopted for selenocysteine (Sec) [45].

### 3.2. Temporal Order of Amino Acids

**Figure 2:**
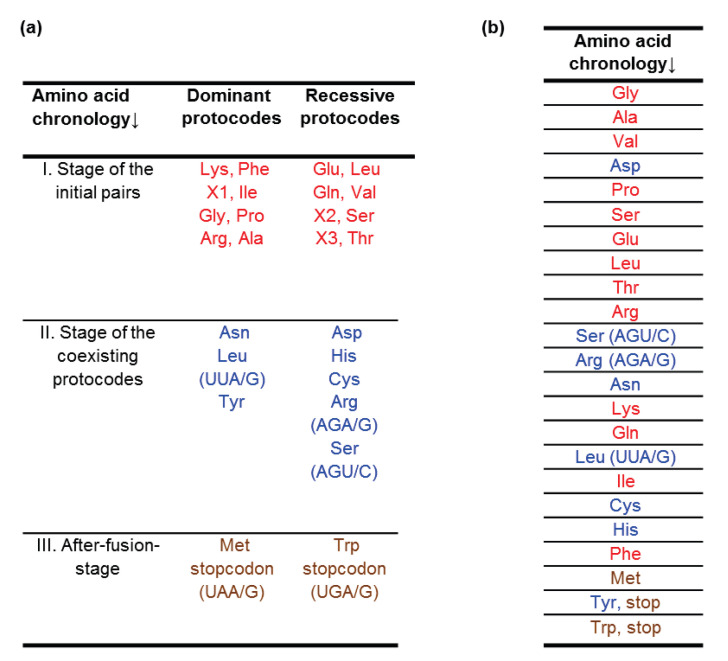
(**a**) Amino acid chronology of the combinatorial fusion cascade; (**b**) amino acid chronology according to the consensus temporal order after Trifonov [46]. Both the combinatorial fusion cascade and the consensus temporal order indicate a later acquisition of the codons UUA/UUG by the amino acid Leu, AGA/AGG by Arg, and AGU/AGC by Ser. As a result, each of these amino acids acquired six codons in the SGC. The color denotes the belonging of the amino acid to the stage of the combinatorial fusional cascade: red—the stage of the initial pairs, blue—the stage of coexisting protocodes, brown—after-fusion-stage.

The combinatorial fusion cascade reveals the principle of the amino acid entry into the genetic code as well as the evolution of triplets. All canonical amino acids (except Trp and Met) were already assigned to their cognate codons in the protocodes’ code before the modern-type translation appeared. In this context, one can compare the temporal order of amino acids from the combinatorial fusion cascade (Figure 2a) with the consensus chronology developed by Trifonov (Figure 2b). This approach is unique because forty different single-factor criteria and multi-factor hypotheses about the chronological order of the appearance of amino acids in the early evolution are summarized in consensus ranking. Such a compact representation is very convenient for our case.

Analyzing the consensus chronology, Trifonov made four conclusions:

1. The first amino acids to have been incorporated in early code were of abiotic origin, namely those that were obtained in classical imitation experiments by S. Miller.
2. In the development of the triplet code, a major role was played by the thermostability of codon–anticodon interactions.
3. New codons appeared in complementary pairs.
4. New codons were simple derivatives of chronologically earlier ones.

The chronology of amino acids in the combinatorial fusion cascade showed good congruence with the consensus chronology according to Trifonov. Exceptions are some cases related to the temporal order, which is based on Miller’s experiment [24]. For example, the negatively charged aspartic acid (Asp) entered the code later than its negatively charged analog glutamic acid (Glu). In contrast, in consensus-time order, Asp is one of the first amino acids in the genetic code. Phenylalanine (Phe) and lysine (Lys) are the initial pair in the dominant protocode, but according to Trifonov, they entered the code much later just before the rare canonical amino acid methionine.

Using only the temperature stability parameter without taking into consideration all the consensus numerous criteria, Trifonov obtained, in particular, the following chronological orders: Arg > Ser (AGU/C) > Cys; Lys > Gln > Leu (UUA/G); Arg (AGA/G) > Trp; His > Met (sign > means here “earlier”). These estimates completely coincide with the time order of the combinatorial fusion cascade. It is worth noticing that the consensus approach and the combinatorial fusion cascade are congruent in the late assignment of AGA and AGG codons to Arg as well UUA and UUG codons to Leu. Within the cascade, this is simply explained by the transition of Arg with the GGG codon from the dominant GC code to the recessive one as well as the transition of Leu with the UUA codon from the recessive AU code to the dominant one (Figure 1).

Trifonov’s consensus principle 3 regarding the complementarity of new codons also finds a simple explanation within the combinatorial fusion cascade. Codon complementarity is a central element of the cascade (see Section 3.3).

Consensus principle 4 about the sequential entry of new codons into code as simple derivatives of chronologically earlier ones leaves room for different interpretations. If it is understood as a modification of a codon by one letter, then it contradicts the third fusion rule (for recessive entities), which allows for the appearance of a new codon with the replacement of bases in the first and third positions. This is where the fundamental difference between the combinatorial fusion cascade and the hypothesis about a gradually evolving code is manifested: The code arose not through progressive evolution, but because of the competition of dominant and recessive entities for the same codons.

### 3.3. Horizontal Transfer of “Complex Elements among the Evolving Entities”

The simulation model of Vetsigian, Woese, and Goldenfeld mentioned in the introduction cannot be directly applied for the combinatorial fusion cascade, since it does not consider the expansion of the code due to the appearance of new bases in triplets. However, it possesses a high degree of generalization, calculating the communal evolution of the competing initially random entities and their genomes in the form of a freely defined codon usage matrix with and without horizontal gene transfer (HGT) [30]. The authors demonstrated that when the HGT is present, the tendency to diversity between the competing entities is reduced and the code tends to achieve near universality.

In the combinatorial fusion cascade, this HGT principle is expressed in an extreme form of the fusion between the recessive and dominant entities. The combinatorial fusion shows how the competing protocodes can be upgraded without destroying their functionality: The original complementarity between small (most of them hydrophobic) and large (hydrophilic) amino acids in all competing protocodes is retained in the SGC. It is obvious for the dominant entities. For example, the codons of Lys and Phe (AAA and UUU) remain complimentary along the entire cascade. In the recessive AU-protocode, for example, Asp and Val lost their initial complementary codons AAU and AUU, but remained complementary in the SCG with new codons GAC and GUC, respectively. An added value from the combinatorial fusion consists of generating new amphiphilic combinations. For example, Lys becomes complementary not only to Phe, but also to hydrophobic Leu from the recessive AU-protocode at the end of the combinatorial fusion cascade.

Amphiphilic amino-acid–RNA complexes could contribute to the compartmentalization that was likely a crucial stage in the emergence of life [47,48,49] and primordial enzymatic functions where amino acids were brought together by non-covalent interactions [50]. Thus, the genetic code generated via the combinatorial fusion cascade could perform other functions before switching to the modern-type translation.

Compartmenting of the ancient protocodes could explain their spatial coexistence, similar to the coexistence of different microorganisms isolated by lipid bilayer. Stueken et al. proposed a study of the origin of life within the global context of the Hadean Earth as a global chemical reactor to benefit from identifying linkages between organic precursors, minerals, and fluids in various environmental contexts [51]. Assuming this approach, the protocodes could emerge independently in different geographic zones. For example, the recessive GC protocode containing Cys could arise in an area with a high concentration of sulfur atoms. An interesting fact of the combinatorial fusion cascade is that the canonic amino acids Lys and its non-canonic derivative Pyl, or structurally similar Sec and Cys are located in the same protocodes, respectively. One of the explanations for this fact is that non-canonical amino acids Pyl and Sec are X1 and X3, which were lost when the spreading across the Earth protocodes merged and then later rediscovered by microorganisms in conditions similar to those where the protocodes appeared [31]. Preiner et al. studying the ancient metabolic pathways of amino acids indicated the existence of the same synthesis pathway for Lys, Pyl, Met, Ile, and Asn [7]. Note that all these amino acids are associated with the dominant AU protocode.

To what extent the combinatorial fusion took place in reality depends on the principal issue: “Why did the combinatorial fusion cascade start with monobasic triplet codons, while only random polymerization of nucleotides could occur on the primitive Earth?”. The answer to this question is not yet known, but may be in the special features of the oligonucleotide replication. P.W. Kudella et al. showed that linking short oligomers from a random sequence pool in a templated ligation reaction significantly reduces the sequence space of product strands [52]. The principles for reducing strand entropy identified in that article are also applicable to non-enzymatic replication.

## 4. Conclusions and Outlook

The concept of a gradually evolving genetic code dominates the science about the origin of the SCG. However, this view is only a hypothesis and cannot be used to negate other approaches such as the combinatorial fusion cascade.

Combinatorial fusion rules provide a simple description of codon assignments based on the fusion of the four protocodes. The corresponding protocodes competed for a limited number of codons. Therefore, dominant and recessive protocodes appeared. It turned out that these competition patterns can be followed up to individual coexisting amino acid pairs. They existed in the initial stage of the combinatorial fusion cascade—the explosion-like transition from prebiotic molecules to the first forms of life.

Along with the mathematical description of the codon assignments, combinatorial fusion cascade explained many features of the standard genetic codes as availability of amino acids with the even and odd numbers of codons, the appearance of stop codons, inclusion of novel canonical amino acids, exceptional high numbers of codons for amino acids arginine, leucinem and serine, and the temporal order of amino acid inclusion into the code.

The time order of amino acids within the cascade demonstrated a good congruence with the consensus time order calculated by Trifonov. The difference between both time orders arose only where the combinatorial fusion cascade contradicted the postulate of the progressive addition of amino acids into the genetic code.

An important property of the combinatorial fusional cascade is the preservation of complementarity between the codons of hydrophobic and hydrophilic canonical amino acids and the generation of new amphiphilic pairs after the fusion of dominant and recessive entities.

The combinatorial fusion cascade broadens the view on biotechnology. As noted at the end of §3, amino acid-modified amphiphilic hairpin pairs as well as replication processes were presumably the driving forces behind the combinatorial fusion cascade. Since the chemical foundations of the functionalization of RNA with amino acids as well as replication processes are well studied and can individually be reproduced in laboratory conditions, there is a possibility of laboratory combination of these processes for an artificial combinatorial fusional cascade. Depending on the set of artificial amino acids for the cascade (which cannot be integrated into proteins) as well as the environment that may be of technical interest, artificial self-replicating entities with the desired biochemical pathways could be developed. Further study of the principles of the combinatorial fusional cascade could help in the search for early life forms on Earth and beyond, and in the long-term, for developing extraterrestrial habitats.
